# Prospective cohort study reveals MMP-9, a neuroplasticity regulator, as a prediction marker of cochlear implantation outcome in prelingual deafness treatment

**DOI:** 10.1007/s12035-022-02732-7

**Published:** 2022-01-21

**Authors:** Monika Matusiak, Dominika Oziębło, Monika Ołdak, Emilia Rejmak, Leszek Kaczmarek, Piotr Henryk Skarżyński, Henryk Skarżyński

**Affiliations:** 1 Oto-Rhino-Laryngosurgery Clinic, Institute of Physiology and Pathology of Hearing, 10 Mochnackiego Street, 02-042 Warsaw, Poland; 2 World Hearing Centre, 17 Mokra Street, 05-830, Nadarzyn, Poland; 3 Department of Genetics, Institute of Physiology and Pathology of Hearing, 10 Mochnackiego Street, 02-042 Warsaw, Poland; 4 Postgraduate School of Molecular Medicine, Medical University of Warsaw, 61 Żwirki i Wigury Street, 02-091 Warsaw, Poland; 5 BRAINCITY, Nencki Institute of Experimental Biology, 3 Pasteura Street, 02-093 Warsaw, Poland; 6 Department of Teleaudiology and Screening, World Hearing Center, Institute of Physiology and Pathology of Hearing, 10 Mochnackiego Street, 02-042 Warsaw, Poland; 7 Heart Failure and Cardiac Rehabilitation Department, 2Nd Faculty of Medicine, Medical University of Warsaw, 8 Kondratowicza Street, 03-242, Warsaw, Poland; 8 Institute of Sensory Organs, 1 Mokra Street, 05-830, Nadarzyn/Kajetany, Poland

**Keywords:** Neuronal plasticity, Congenital deafness, Cochlear implantation, MMP-9

## Abstract

Because of vast variability of cochlear implantation outcomes in prelingual deafness treatment, identification of good and poor performers remains a challenging task. To address this issue, we investigated genetic variants of matrix metalloproteinase 9 (*MMP9*) and brain-derived neurotrophic factor (*BDNF*) and plasma levels of MMP-9, BDNF, and pro-BDNF that have all been implicated in neuroplasticity after sensory deprivation in the auditory pathway. We recruited a cohort of prelingually deaf children, all implanted before the age of 2, and carried out a prospective observation (*N* = 61). Next, we analyzed the association between (i) functional *MMP9* (rs20544, rs3918242, rs2234681) and *BDNF* (rs6265) gene variants (and their respective protein levels) and (ii) the child’s auditory development as measured with the LittlEARS Questionnaire (LEAQ) before cochlear implant (CI) activation and at 8 and 18 months post-CI activation. Statistical analyses revealed that the plasma level of MMP-9 measured at implantation in prelingually deaf children was significantly correlated with the LEAQ score 18 months after CI activation. In the subgroup of DFNB1-related deafness (*N* = 40), rs3918242 of *MMP9* was significantly associated with LEAQ score at 18 months after CI activation; also, according to a multiple regression model, the ratio of plasma levels of pro-BDNF/BDNF measured at implantation was a significant predictor of overall LEAQ score at follow-up. In the subgroup with DFNB1-related deafness, who had CI activation after 1 year old (*N* = 22), a multiple regression model showed that rs3918242 of *MMP9* was a significant predictor of overall LEAQ score at follow-up.

**Supplementary Information:**

The online version contains supplementary material available at 10.1007/s12035-022-02732-7.

## Introduction

### Congenital Deafness Treatment with Cochlear Implantation and Variability of Its Outcome

Congenital deafness is a disabling condition which, untreated, has far-reaching consequences and profoundly affects the quality of the patient’s life. Cochlear implants (CIs) and neuroprostheses commonly used in treating the disease are a very effective tool for restoring absent auditory function. However, despite the undeniable success of the method, one caveat is a large degree of variability in outcomes across individual CI users. Beside the very good performers, who develop speech and language almost normally, some implanted children, despite great effort at rehabilitation, never reach age-appropriate proficiency in speech, language, and verbal communication [1–3]. For clinicians, it is difficult to preoperatively predict how well an individual patient will perform with a CI. Only a part of the variance in performance can be accounted for by known factors, such as age of implantation, etiology of hearing loss, existence of comorbidities, and others [1–12].

Genetic causes of hearing loss are heterogeneous, and there are ethnic-specific differences in the involvement of particular genes in its development. In the majority of countries, mutations in the *GJB2* and *GJB6* genes (DFNB1 locus) are the leading cause of hearing loss, and they are identified in up to 50% of patients with severe-to-profound autosomal recessive nonsyndromic deafness [13]. The presence of pathogenic variants in the DFNB1 locus causes malfunctioning of the organ of Corti, although the molecular mechanism behind it is still under investigation [14, 15]. Delivery of electrical stimulation to the auditory pathway involves an interplay between implant software and brain tissue (wetware) [1, 4]. For this reason, it is thought that the missing factors contributing to CI outcome, which might also serve as either biochemical or genetic biomarkers of auditory development following a CI, could be located in the biological environment of the implant [4]. Finding such biomarkers would be important, as it would allow increased efforts to be made in identifying children at risk of failure of their CI.

### Neuronal Plasticity

The cerebral cortex of a developing child responds to sensory stimuli coming from the child’s environment by undergoing neuronal plasticity [1, 16]. The ability to modify the strength and efficacy of cortical synapses is the neurons’ essential attribute and is needed for learning and memory, but in an aberrant form it contributes to many pathological conditions such as addiction, schizophrenia, or epilepsy [1, 17–19]. The recent introduction of the tetrapartite synapse concept has drawn attention to the extracellular matrix (ECM) as a factor contributing to synapse function and dysfunction [17]. The ECM might be cleaved by matrix metalloproteinases (MMPs), making it particularly susceptible to remodeling processes [17, 18, 20–23]. MMPs are a family of metzincin proteinases with an established role in developmental plasticity -well documented in the case of MMP-9 [20, 21]. Experimental data demonstrate that MMP-9 is a critical factor in late-phase long-term potentiation (LTP), which is considered the physiological basis of synaptic plasticity [24–27]. It has also been postulated, based on research on rodent models, that MMP-9 is involved in plasticity during critical periods in development by regulating synaptogenesis, axonal growth, and myelination [20]. Furthermore, genetic association studies have supported the role of MMP-9 in a series of brain disorders involving aberrant plasticity [17, 19, 28]. BDNF is another protein of pivotal importance in neuronal plasticity [29, 30]. The molecule can potentiate synaptic transmission, thus inducing LTP, and is tightly connected with cognitive processes, memory, and learning [29, 31, 32]. Interestingly, the protein might be cleaved from pro-BDNF into a mature BDNF form (BDNF) by MMP*-*9 [29, 32–36].

Given the reported role of both MMP-9 and BDNF in neuronal plasticity, it may be beneficial to study their roles in auditory plasticity after a child with congenital deafness receives a CI. For this reason, we have designed an association study between polymorphisms known to affect *MMP9* and *BDNF* expression and auditory development as measured by the LEAQ score. The study was performed in a group of deaf infants and toddlers implanted with the same type of device. We have also searched for associations among plasma levels of MMP-9, BDNF collected at cochlear implantation, and pro-BDNF/BDNF ratio and the children’s auditory development.

### Aim of the Study

In this study, we wanted to test the hypothesis that carrying a specific set of functional *MMP9* and *BDNF* gene variants, and protein plasma levels of MMP-9, BDNF, and pro-BDNF/BDNF ratio, measured at cochlear implantation, can serve as prognostic biomarkers of functional outcomes of CI treatment in a population of congenitally deaf children. To verify this assumption, we collected a dataset from a cohort of 70 implanted infants and toddlers. To our knowledge, the role of *MMP9* and *BDNF* genes together with their proteins in the neuroplasticity of the human auditory system has not been explored so far.

## Material and Methods

### Study Design, Participants, and Ethical Approval

This prospective cohort study was done between December 2016 and December 2019 in the Institute of Physiology and Pathology of Hearing in Warsaw, Poland. We recruited infants and toddlers with hearing loss, who underwent cochlear implantation with the same type of device, performed by the same surgeon, and who all had their speech processor activated before the age of 2. Inclusion criteria were congenital bilateral profound sensorineural hearing loss, confirmed by auditory brainstem responses (ABR). Exclusion criteria were the presence of any acute inflammation confirmed by CRP (C-reactive protein) measurements and the presence of environmental risk factors, such as chronic concomitant disease, severe prematurity, asphyxia, or history of viral infection during pregnancy. After activation of the CI, parents or caregivers followed instructions of auditory–verbal therapy. Children were clinically assessed for auditory development by using the LEAQ before CI activation and at the 8^th^ and 18^th^ month after CI activation (LEAQ_0, LEAQ_8, LEAQ_18). Additionally, at cochlear implantation patients had their blood sampled for genetic polymorphisms testing and MMP-9, BDNF, pro-BDNF, and CRP plasma level (MMP-9_0, BDNF_0, pro-BDNF_0). Out of 70 children enrolled in the study, one patient was excluded due to autism spectrum disorder diagnosed during follow-up (and therefore assessed as having unreliable auditory development); two cases were excluded due to the parents withdrawing from the study; and six patients were excluded due to elevated CRP levels. LEAQ scores were successfully collected from all participants at all three intervals. Demographic data were also obtained. Participants underwent genetic testing of the DFNB1 locus and were classified into two subgroups: DFNB1-related deafness and not DFNB1-related deafness*.* Following the line of maximal homogeneity, patients with DFNB1-related deafness were divided into subgroups according to their age at CI activation: “CI activation before 1 year old” and “CI activation after 1 year old”.

### Auditory Development Assessment

Participants were assessed for their auditory development by the LEAQ, which is designed for very young children [37]*.* LEAQ consists of 35 questions with a “yes” or “no” answer. The total score is the number of “yes” answers. The LEAQ has been validated in over 20 languages [38–43].

### Polymorphism Selection and Genotyping

Genotyping of functional variants of *MMP9* and *BDNF* was included to the study protocol. We selected variants that exert a documented effect on gene expression and were associated with different clinical conditions.

The rs3918242 and rs2234681 polymorphisms are situated in the *MMP9* gene promoter and may affect its expression [17, 19, 22, 28]. The rs3918242 of *MMP9* gene has been found to be associated with depression and schizophrenia, with the C allele increasing the susceptibility risk to these disorders, and the T allele, on the other hand reducing the risk [17, 28]. The rs2234681, a microsatellite repeat of (CA)n, is a multiallelic polymorphism. The length of the CA repeat is closely related to *MMP9* transcriptional activity. It increases the binding affinity of the transcription factor(s) with the *MMP9* promoter [44–46]. This polymorphism has been associated with cardiac and kidney problems [47, 48]. The rs20544 is located in the 3′UTR of *MMP9* and is strongly associated with the severity of a chronic delusional syndrome in schizophrenia patients [49]. The rs6265 of *BDNF* gene results in a valine-to-methionine substitution (Val66Met), leading to reduced *BDNF* expression. This polymorphism has been widely implicated in a variety of psychiatric disorders such as unipolar and bipolar disorder, depression, and anxiety [30].

The *MMP9* polymorphism rs3918242 (NM_004994.2:c.-1590C > T) was genotyped using the PCR–RFLP method. The genomic region encompassing rs3918242 was amplified using forward 5′-GCCTGGCACATAGTAGGCCC-3′ and reverse 5′-CTTCCTAGCCAGCCGGCATC-3′ primers (Oligo IBB PAN, Warsaw, Poland), and the PCR product was digested with the PaeI restriction enzyme (Thermo Fisher Scientific, Waltham, MA, USA). The allele containing reference variant C was represented by a DNA band of size 435 bp, and the allele containing alternative variant T was represented by bands of sizes 188 and 247 bp.

Genotyping of *MMP9* polymorphism rs2234681 (13–26 CA repeats around NM_004994.2:c.-90) included amplification with forward 5′-FAM-CTGAGGGCCTGCGGTTTCCT3′ and reverse 5′CCTTGACAGGCAAGTGCTGACT3′ primers (Oligo IBB PAN). PCR products were separated by capillary electrophoresis on a 3500xL Genetic Analyzer (Applied Biosystems, Foster City, CA, USA). Results were analyzed with GeneMapper v4.1 software (Applied Biosystems) and reported as either less than 20 CA repeats (< 20) or equal to or more than 20 CA repeats (≥ 20).

The *MMP9* polymorphism rs20544 (NM_004994.2:c.*3C > T) and the *BDNF* polymorphism rs6265 (NM_170735.5:c.196G > A) were genotyped using predesigned TaqMan SNP genotyping assays (Applied Biosystems) and a real-time PCR system (Viia7, Thermo Fisher Scientific).

The accuracy of genotyping was confirmed by Sanger sequencing in randomly selected samples. The results were 100% concordant. Detailed genotyping conditions are available upon request.

### Plasma Sample Collection

Blood samples were collected on heparin and centrifuged at 1400 g for 15 min. Next, plasma was obtained, aliquoted, and stored at − 80 °C for further analysis. Total protein content was measured with a BCA protein assay kit (Thermo-Scientific) following the manufacturer’s protocol.

### MMP-9, BDNF, and pro-BDNF Plasma Level Measurements

Plasma concentrations of MMP-9, BDNF, and pro-BDNF were determined by ELISA (MMP-9 and BDNF – R&D Systems Inc., Minneapolis, USA; pro-BDNF – Aviscera Bioscience, Santa Clara, CA, USA) according to the manufacturer’s protocol. A total of 30 μg/μl of protein from each plasma sample was diluted 70-fold (MMP-9) or 20-fold (BDNF and pro-BDNF) with calibration diluent from the assays and analyzed in duplicate. The optical density of wells was measured at 450 nm using an automated microplate reader (Sunrise Microplate Absorbance Reader).

### Statistical Analyses

#### Paired Comparisons Methodology

For all tested follow-up intervals (i.e., at the time of CI activation, and at the 8^th^ and 18^th^ month after CI activation), comparisons of mean LEAQ scores, BDNF levels, and MMP-9 levels were made between patients with different genotypes using a Welch two-sample *t*-test (if test assumptions were met) or the Wilcoxon rank-sum test. All calculations were performed with R (version 3.6.3). Results were considered statistically significant at a *p*-value ≤ 0.05.

#### Correlation Analysis Methodology

LEAQ scores measured at different time intervals from CI activation and BDNF, MMP-9 levels, and pro-BDNF/BDNF ratios measured at CI activation were tested for correlation and strength using a Pearson test (if test assumptions were met) or a Spearman test. Prior to correlation tests, a Shapiro–Wilk test of normality was made in order to check assumptions. All variables for which the correlation was tested were normalized using the min–max scaling method. Correlations were considered statistically significant at *p*-value ≤ 0.05. All computations were made using R version 3.6.3 (2020).

#### Modelling Methodology

To address the longitudinal aspect of the study design, linear mixed-effect models were built*.* BDNF, MMP-9, and pro-BDNF/BDNF ratio levels, as well as *BDNF* rs6265, *MMP9* rs3918242, MMP9 rs20544, and *MMP9* rs2234681 genotypes, sex, follow-up interval, and age at CI activation were included in the models as a set of predictors. Differences were considered statistically significant at *p* ≤ 0.05. All calculations were performed with R (version 3.6.3) and lme4, blme, stargazer, and lmerTest packages. Further details about the methodology can be found in our previous publication [44].

## Results

### Sample Demographics and Auditory Development

In the group of 61 implanted children, 28 (45.9%) were girls and 33 boys (54.1%). In 24 children (39.3%) some responses at or over 80 dB on ABR were recorded, while in 37 children (60.7%) there were no responses on ABR. The mean age at CI activation in the study group was 411.4 days (min = 208; max = 739; SD = 135.1). In 40 cases, DFNB1-related deafness was identified. In this subgroup, 18 (45%) were girls and 22 were boys (55%). In 14 children (35.0%), some responses on ABR at or over 80 dB were recorded, while in 26 children (65.0%) there were no responses on ABR. The mean age at CI activation in the subgroup was 407.7 days (min = 208, max = 654, SD = 128). All children were implanted with the Med-El Synchrony CI and became regular CI users. All participants were of Caucasian origin.

### Genotyping

Distributions of genotypes were in the Hardy–Weinberg equilibrium in the whole studied cohort. For rs3918242 of *MMP9* gene, the C/C genotype was found in 43 cases (70.49%) and the C/T genotype in 18 cases (29.51%). For rs20544 of *MMP9* gene, the C/C genotype was found in 16 cases (26.23%), the C/T genotype in 29 cases (47.54%), and the T/T genotype in 16 (26.23%) cases. For rs2234681 of *MMP9* gene, the < 20/ < 20 genotype was found in 17 cases (27.87%), the < 20 ≥ 20 genotype in 34 cases (55.74%), and the ≥ 20/ ≥ 20 genotype in 10 cases (16.39%). For rs6265 of *BDNF* gene, the Val/Met genotype was found in 18 cases (29.51%) and the Val/Val genotype in 43 cases (70.49%). Allele frequencies of the tested variants did not differ statistically from those reported in population databases (Supplementary Table 1).

### MMP-9, BDNF, and pro-BDNF Plasma Levels

The mean value of protein plasma level of MMP-9_0 was 236.94 ng/ml, and the levels varied from 31.14 to 769.67 (SD 135.59). Mean value of protein plasma level of BDNF_0 was 2.28 ng/ml, the levels varied from 0.25 to 12.17 (SD 1.91). Mean value of protein plasma level of pro-BDNF_0 was 19.31, and the levels varied from 0.00 to 162.16 (SD 36.08). Mean value of pro-BDNF_0/BDNF_0 ratio was 15.02, and the values varied from 0.00 to 161.55 (SD 31.64).

### Analyses in the Study Group

In the study group, no significant associations between *MMP9* and *BDNF* genetic variants and LEAQ score at any of the tested follow-up intervals (before CI activation, and at the 8^th^ and 18^th^ month after CI) were identified (data not shown). No significant associations were identified between pre-CI ABR results and LEAQ score at any of the tested follow-up intervals, as well as between pre-CI ABR results and *MMP9* and *BDNF* genetic variants (data not shown).

**Fig. 1:**
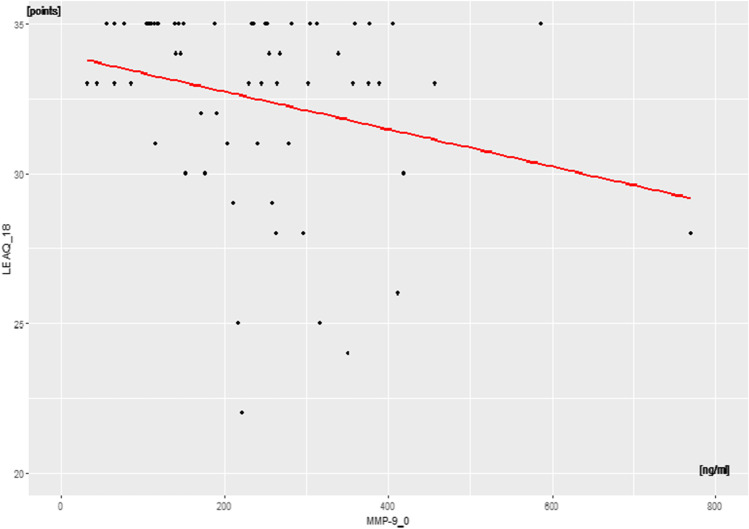
Correlation between plasma levels of MMP-9_0 and auditory development measurements at the 18^th^ month after CI activation (LEAQ_18) in the study group

We did not observe any significant correlations of the protein levels of MMP-9_0, BDNF_0, and pro-BDNF_0/BDNF_0 ratio with LEAQ_0 and LEAQ_8. Testing correlations between MMP-9_0, BDNF_0, and pro-BDNF_0/BDNF_0 ratio with LEAQ_18 showed a weak negative correlation between MMP-9_0 and LEAQ_18 score (*p* < 0.05, rho = –0.25) (Fig. 1).

### Analyses in the Subgroup with DFNB1-Related Deafness

In the subgroup with DFNB1-related deafness, we identified a significant association between rs3918242 *MMP9* and LEAQ 18. No statistically significant associations were seen for the other tested polymorphisms at any of the follow-up intervals (Table 1). No significant associations were identified between pre-CI ABR results and LEAQ score at any of the tested follow-up intervals, as well as between pre-CI ABR results and *MMP9* and *BDNF* genetic variants (data not shown).

**Table 1:** Association of auditory development measures (LEAQ score) with *MMP9* and *BDNF* variants in all tested intervals in the subgroup with DFNB1-related deafness (*N* = 40). * indicates *p* < 0.05

	Follow-up interval	Mean LEAQ score (SD)	*p*-value
*MMP9* rs3918242			
C/C(30) C/T(10)	0	6.5 (7.1) / 2.5 (3.9)	0.06
C/C(30) C/T(10)	8	27.8 (4.6) / 25.3 (8.3)	0.5
C/C(30) C/T(10)	18	33.2 (2.7) / 30.6 (3.9)	0.03*
*MMP9 *rs2234681			
< 20/ < 20 (11) ≥ 20/ ≥ 20(6)	0	7.1 (9) / 7.3 (8)	0.7
< 20/ < 20 (11) ≥ 20/ ≥ 20(6)	8	29.5(3.4) / 25.8 (6.2)	0.2
< 20/ < 20 (11) ≥ 20/ ≥ 20(6)	18	33 (2.9) / 31.7 (4.4)	0.7
< 20/ < 20 (11) < 20/ ≥ 20 (23)	0	7.1 (9) / 4.3 (4.9)	0.4
< 20/ < 20 (11) < 20/ ≥ 20 (23)	8	29.5 (3.4) / 26.4 (6.4)	0.2
< 20/ < 20 (11) < 20/ ≥ 20 (23)	18	33 (2.9) / 32.6 (3.1)	1.0
< 20/ ≥ 20 (23) ≥ 20/ ≥ 20(6)	0	4.3 (4.9) / 7.3 (8)	0.2
< 20/ ≥ 20 (23) ≥ 20/ ≥ 20(6)	8	26.4 (6.4) / 25.8 (6.2)	0.8
< 20/ ≥ 20 (23) ≥ 20/ ≥ 20(6)	18	32.6 (3.1) / 31.7 (4.4)	0.8
*MMP9* rs20544			
C/T(21) T/T(11)	0	4.7 (4.9) / 8.5 (9.1)	0.3
C/T(21) T/T(11)	8	26.5 (6.2) / 29.6 (3.4)	0.4
C/T(21) T/T(11)	18	32.62 (3.1) / 33.3 (2.9)	0.3
C/C(8) T/T(11)	0	3.6 (6.3) / 8.5 (9.1)	0.2
C/C(8) T/T(11)	8	25.6 (6.7) / 29.6 (3.4)	0.1
C/C(8) T/T(11)	18	32.5 (3.1) / 33.3 (2.9)	1.0
C/C(8) C/T(21)	0	3.6 (6.3) / 4.7 (4.9)	0.6
C/C(8) C/T(21)	8	25.6 (6.7) / 26.5 (6.2)	0.7
C/C(8) C/T(21)	18	32.5 (4) / 32.2 (3.1)	0.4
*BDNF* rs6265			
Val/Val (29) Val/Met (11)	0	4.4 (5.4) / 8.4 (9)	0.1
Val/Val (29) Val/Met (11)	8	27.1 (5) / 27.4 (7.7)	0.4
Val/Val (29) Val/Met (11)	18	32.7 (3) / 32.2 (3.7)	0.8

There was no correlation between plasma levels of MMP-9_0 and BDNF_0 and pro-BDNF_0/BDNF_0 ratio with LEAQ_0 and LEAQ_8 in the DFNB1-related deafness subgroup.

**Fig. 2:**
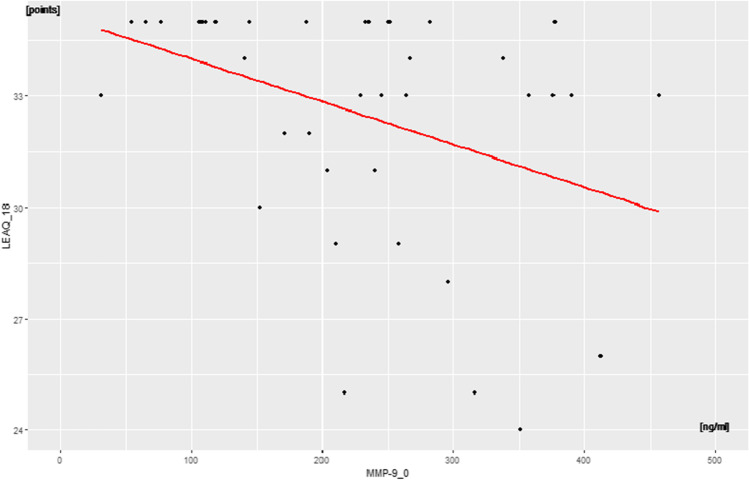
Correlation between MMP-9_0 plasma level and LEAQ score at the 18^th^ month after CI-activation (LEAQ_18) in the DFNB1-related deafness subgroup

A weak negative correlation was found between plasma levels of MMP-9_0 with LEAQ_18 in the DFNB1-related deafness subgroup (*p* < 0.01, rho = –0.4) (Fig. 2)*.* There was no correlation between BDNF_0 and pro-BDNF_0 /BDNF_0 ratio and LEAQ_18.

To build a linear mixed-effect model using plasma levels of the analyzed proteins as predictors for the subgroup with DFNB1-related deafness, observations for every patient were broken down into single measurements of language development (LEAQ score) from CI activation to the 18^th^ month after CI activation. This gave a set of 120 observations. The model revealed that, apart from the follow-up interval, the pro-BDNF_0/BDNF_0 ratio is a significant predictor of auditory development. The average *R*^2^ for the model was 0.72, indicating that it could explain a considerable level of variation in the data. The *p*-values of the predictor variables and their associated impact on global outcome scores are shown in Table 2. In a similar way, for the same subgroup we built a multiple regression model using the tested polymorphisms as predictors, and the model revealed that one significant predictor of auditory development was follow-up interval (data not shown).

**Table 2:** Summary of linear mixed-effect model for LEAQ score in the DFNB1-related deafness subgroup. Estimates show the impact of significant predictors (marked by asterisks). *R*^2^ = 0.72. SE, Standard Error

Predictors	Dependent variable: LEAQ, (SE)	*p*-value
Follow-up interval (months)	1.465***(0.086)	< 0.001
Sex	0.863 (1.301)	0.508
BDNF_0	0.245 (0.348)	0.483
MMP9_0	–0.008 (0.007)	0.256
Ratio pro-BDNF_0/BDNF_0	–0.049*(0.024)	< 0.05
Age at CI activation	0.008 (0.006)	0.183
Constant	7.385*(2.936)	< 0.05
Observations	120	
Log likelihood	–411.014	
Akaike info criterion	840.028	
Bayesian info criterion	865.115	

To give a clinical interpretation of the impact of the significant predictor pro-BDNF_0/BDNF_0 ratio on auditory development, the regression coefficient shows that a decrease in the ratio of 20 (1:0.05) will, on average, result in an increase of LEAQ score of 1 point in an implanted child with DFNB1-related deafness.

### Analyses in Subgroups: DFNB1-Related Deafness and CI Activation Before and After 1 Year

In none of the two DFNB1-related subgroups (CI < 1 or CI > 1 y. o.) did we find any significant association between the analyzed *MMP9* and *BDNF* genetic variants and LEAQ score at any of the tested follow-up intervals (data not shown).

**Fig. 3:**
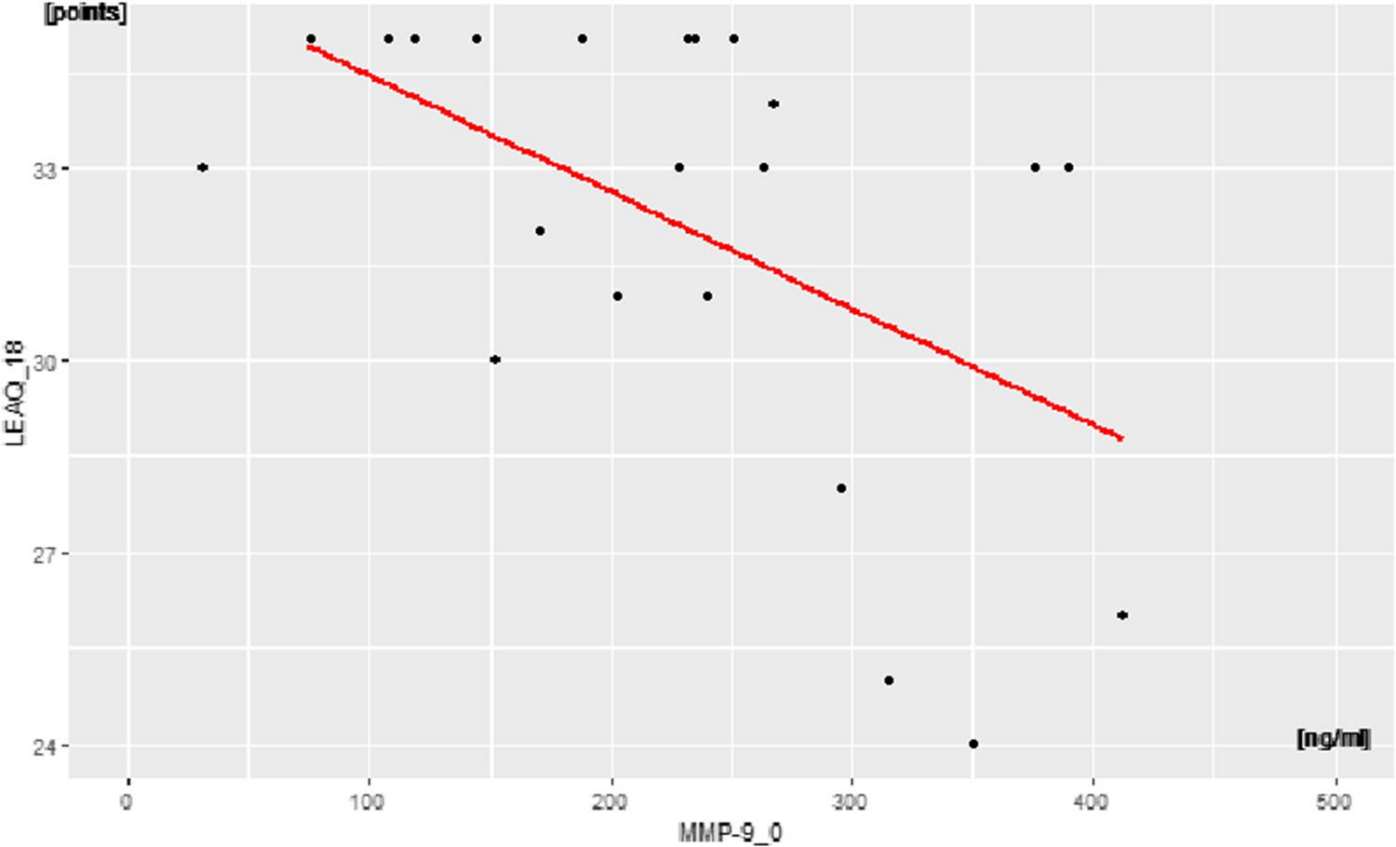
Correlation between MMP-9_0 and auditory development measurements at 18 months after CI activation (LEAQ_18) in the subgroup with DFNB1-related deafness and CI activation after 1 year old

In the subgroup with DFNB1-related deafness and CI activation after 1 year, hereafter referred to as older, no correlation between MMP-9_0 and LEAQ_0 was found, a positive correlation between BDNF_0 and LEAQ_0 score (p = 0.03, Rho = 0.4) and a negative correlation between pro-BDNF_0/BDNF_0 ratio and LEAQ_0 score (*p* = 0.01, Rho = –0.5) were found. Testing between MMP-9_0, BDNF_0, and pro-BDNF_0/BDNF_0 ratio with LEAQ_8 did not show any correlation. For LEAQ_18, it showed a moderate negative correlation between MMP-9_0 and LEAQ_18 score (*p* = 0.01, rho = –0.5) (Fig. 3) and no correlation between BDNF_0 and pro-BDNF_0/BDNF_0 and LEAQ_18.

Similar analyses performed in the DFNB1-related deafness and CI activation up to 1 year subgroup, hereafter referred to as younger, did not reach statistical significance.

For both younger and older subgroups, we built a linear mixed models on a set of 54 and 66 observations, respectively, with the tested polymorphisms as predictors*.* The model built for the older subgroup revealed that a significant predictor of auditory development outcome (LEAQ score), apart from the follow-up interval, is rs3918242 of *MMP9* (Table 3). The regression coefficients show that an average patient who is a carrier of a C/C variant of rs3918242 of MMP9 will score, on average, 6.49 points higher at any interval than a carrier of a C/T variant. The *R*^2^ for the model was 0.7. The *p*-values of the predictor variables and their associated impact on global outcome scores are shown in Table 3. For the younger group, the model with tested polymorphisms as predictors revealed that the only significant predictor is the follow-up interval (data not shown). On the same sets of observations, we built multiple regression models for both subgroups, using inclusion of plasma levels of analyzed proteins (measured at CI activation) as predictors. The models did not reveal statistical significance for any of the predictors, apart from follow-up interval (data not shown).

**Table 3:** Summary of linear mixed-effect model for LEAQ score in the subgroup with DFNB1-related deafness and CI activation after 1 year. Estimates show the impact of significant predictors (marked by asterisks). *R*^2^ = –0.7. SE, standard error

Predictors	Dependent variable: LEAQ, (SE)	*p*-value
Follow-up interval (months)	1.346***(0.121)	< 0.001
Sex	0.231 (2.100)	0.913
*BDNF* rs6265	1.931 (2.330)	0.410
*MMP9* rs2234681 = < 20, ≥ 20	5.390 (5.746)	0.352
*MMP9* rs2234681 = ≥ 20, ≥ 20	–1.271 (3.684)	0.731
*MMP9* rs20544	–8.156 (5.286)	0.128
*MMP9* rs3918242	–6.494 (2.830)*	< 0.05
Age at CI activation	–0.001 (0.010)	0.929
Constant	13.209*(5.963)	< 0.05
Observations	66	
Log likelihood	–212.487	
Akaike info criterion	446.974	
Bayesian info criterion	471.060	

We have determined relations between mean values of plasma levels of the tested proteins and genetic variants. We did not find any statistically significant differences either in the study group or in the DFNB1-related deafness subgroup.

## Discussion

The present report seems to be the first to identify molecular and genetic biomarkers of neuronal plasticity after restoration of an absent sensory function in humans. The study has investigated possible associations between functional variants of the *MMP9* and *BDNF* genes (and their products in plasma) and auditory development in congenitally deaf children after cochlear implantation. Our main findings can be summarized as follows. (i) Plasma level of MMP-9 protein measured at cochlear implantation in congenitally deaf children is significantly correlated with auditory development measurements (LEAQ score) at 18 months after the device activation. (ii) In the subgroup with DFNB1-related deafness, functional *MMP9* variant rs3918242 significantly impacts auditory development measured at the 18^th^ month after the device activation; it is also a significant predictor of overall LEAQ score in the subgroup with DFNB1-related deafness and activation after at the 1 year. (iii) The pro-BDNF/BDNF ratio measured at cochlear implantation is a significant predictor of auditory development (LEAQ score) as measured in the subgroup with DFNB1-related deafness.

For this prospective cohort study, we enrolled children diagnosed with bilateral congenital deafness without any comorbidities (and with noncontributory medical and pregnancy history). Patients were implanted with the same type of device, by the same experienced surgeon, and had their speech processor activated before second birthday; they also received the same rehabilitation program and their auditory development was observed for 18 months after CI activation. The literature already indicates that etiological homogeneity, such as mutations in the DFNB1 locus, has a predictive value for cochlear implantation outcome [6, 7, 10–12]. Following this lead, our analyses focused on the subgroup of children with DFNB1-related deafness, which was further divided into patients with CI activated before and after 1 year of life.

### Functional Variant rs3918242 of the *MMP9* Gene and Auditory Development

Out of the tested genetic variants of *MMP9* and *BDNF*, only rs3918242 *MMP9* showed significant association with auditory development in the subgroup with DFNB1-related deafness. At the 18^th^ month after CI activation, carriers of C/C genotype scored, on average, 2.6 points higher than carriers of the C/T genotype (Table 1). This result is in line with results of our previous retrospective study, which reported the contribution of this polymorphism to LEAQ score over a 24-month follow-up in a cohort of 100 children with DFNB1-related deafness [50]. In the retrospective study, carriers of the C/C genotype scored higher than carriers of the C/T genotype, although the differences between their mean LEAQ scores did not reach significance. In the current analysis, we found statistically significant differences between mean LEAQ scores reached by the C/C and C/T genotype carriers at 18-month follow-up. Since there is no literature on the molecular regulation of neuroplasticity after deafness treatment, our results of testing associations of *MMP9* polymorphisms need to be read in the context of published data on how these polymorphisms behave in other clinical conditions involving neuroplasticity. We observed a cohort of CI children, who, after activation of the CI and in response to stimulation delivered by the device, underwent a process of dynamic remodeling of cortical neuronal connections. The role of rs3918242 of *MMP9* in neuronal plasticity has only been studied so far in the context of aberrant plasticity, e.g., in schizophrenia or addictions [28, 49, 51–53]. Of note, several groups have reported inconsistent data in case–control studies of rs3918242 of *MMP9* involvement in the clinical picture of these diseases. For example, Rybakowski et al. [28] reported that the C/C genotype of rs3918242 of *MMP9* was implicated in schizophrenia susceptibility, but this finding has not been reproduced in other schizophrenia studies [51, 52]. However, a role for this polymorphism in schizophrenia has been confirmed in modifying the relationship between clinical severity and certain environmental risk factors [19]. Additionally, in an ethanol addiction study reported by Samochowiec et al. [53], the authors showed that the C/C genotype was more frequent in the families of alcoholics than in controls*.*

In our material, the impact of rs3918242 of *MMP9* on auditory development, among other genetic and clinical factors, was seen in a multiple regression model built around the older subgroup. The model indicated the significant effect of rs3918242 of *MMP9* on overall LEAQ score at follow-up (Table 3). For the average carrier of the C/C genotype, the average estimated LEAQ score was found to be higher by 6.49 points than for a carrier of the C/T genotype, a difference which can be converted into 7.7 months of delay in auditory development after cochlear implantation [38]. Clearly, the effect of follow-up interval has the highest impact on LEAQ score as predictor in the model, since it reflects the duration of CI use and the biological age of the implanted child, so it will inevitably rise from one follow-up interval to the next. It has already been well documented that the child’s age when auditory development is measured is the strongest factor in shaping the outcome [2].

### Plasma Level of MMP-9 Protein Measured at Cochlear Implantation

In our material, plasma samples taken at cochlear implantation from carriers of less transcriptionally active C/C genotype of rs3918242 of *MMP9* showed lower levels of MMP-9 protein than samples from carriers of the more active C/T genotype. This pattern was repeated in the DFNB1-related deafness subgroup, although these differences did not reach statistical significance (*p* = 0.07 and *p* = 0.06, respectively) (Supplementary Tables 2 and 3)*.* Further analyses showed a significant negative correlation between the plasma level of MMP-9 protein measured at cochlear implantation with LEAQ score measured at the end-point observation (LEAQ_18) for (i) the whole study group; (ii) the DFNB1-related deafness subgroup; and (iii) the subgroup with DFNB1-related deafness and CI activation after 1 year of life (*p* = 0.04, rho = –0.25; *p* = 0.005, rho = –0.4; and *p* = 0.01, rho = 0.5, respectively) (Figs. 1–3). These correlations indicate that a deaf child, who at cochlear implantation has a lower MMP-9 protein plasma level is predisposed to better auditory outcome after 18 months of speech and language rehabilitation. Only for the youngest group do we not see this trend.

Rs3918242 of *MMP9*, being a result of functional C > T transition in the gene promoter region, exerts a critical effect on transcriptional activity [51, 52]. This finding may be interpreted in line with data reported in our retrospective study, in which we did not find a significant association between rs3918242 of *MMP9* and auditory development for children implanted before their first birthday, contrary to children implanted after their first birthday [50]. However, this finding needs to be regarded with caution, since differences in MMP-9 protein plasma levels for carriers of the C/C and C/T genotypes did not reach significance. On the other hand, this age-dependent difference also supports our other conclusion that there may be differences in molecular mechanisms underlying cognitive processing in very young children (implanted before and after their first birthday). Data reported in this paper suggest that activated pathways may not involve MMP-9 if the delivery of sensory stimuli takes place before the first year of life. Plasma levels of MMP-9_0 and BDNF_0 proteins were not significantly affected by the other functional variants mentioned (Supplementary Tables 2 and 3)*.*

To date, we have not found any published protein data for plasma samples taken from a homogenous cohort of congenitally sensory-deprived patients. For this reason and to gain more insight into the molecular background of neural plasticity, we need to compare our results with reports from studies involving other physiological and pathological conditions. The role of MMP-9 in neuronal plasticity has already been well-studied, and its influence on this process has been documented in memory and learning and various pathological neuropsychiatric conditions, such as schizophrenia, addiction, or epilepsy [17, 18, 51, 54]. A body of literature describes, for rodents, an important role for the MMP-9 protein in the critical plasticity period [20]. This molecule is mainly involved in the active structural and functional reorganization of excitatory synapses and dendritic spines and is subject to multifactorial regulation at several levels, such as gene expression, mRNA stability, and localization (including translocation toward dendrites and synapses), protein production and release, and enzymatic activity [17, 18, 20, 22, 49, 55]. Expression studies of *MMP9* in auditory pathway, apart from the cortex, are sparse and limited only to animal data. The expression of *MMP9* has been detected in the organ of Corti, specifically in the pillar cells and inner and outer hair cells, and in the spiral ganglion cells [56–59]. Studies on *MMP9* knockout mice have shown that the MMP-9 protein is critical for appetitive learning and memory formation [60]. Research on human schizophrenia patients shows that elevated MMP-9 protein levels are significantly correlated with cognitive decline, particularly in terms of language, fluency, and verbal and general memory [51, 54, 61, 62].

Our data do not show the actual, temporal relation between MMP-9 protein levels in plasma and the phenotype. Changes in MMP-9 plasma levels may influence the phenotype. This has been indirectly observed in animal models, as well as in clinical studies on Fragile X syndrome (FXS) children treated with the MMP-9 inhibitor minocycline [63–65]. FXS is due to a lack of FMRP (Fragile X Mental Retardation Protein), which is associated with alterations in the expression of MMP-9, and in animal models there are elevated levels of the protein in the hippocampus, which is lowered after minocycline administration. Early treatment (before the fourth week of life) with minocycline promotes the maturation of dendritic spines in vivo and in vitro and relieves anxiety and improves cognition [63]. In human trials, authors report significant decreases in typical symptoms of FXS after minocycline administration (attention deficits, mood disorders, hyperactivity, cognition, and language fluency) [64].

### Ratio of pro-BDNF/BDNF Plasma Levels Measured at Cochlear Implantation

In light of our current state of knowledge about the molecular background of neural plasticity and the role of BDNF in these processes, the apparent lack of involvement of BDNF in the results presented here is surprising [29, 30, 66, 67]. We have found no significance of *BDNF* polymorphisms, nor a role for the protein in auditory development, and no correlation between pro-BDNF_0/BDNF_0 ratio and LEAQ scores. Nevertheless, a multiple regression model based on data from the DFNB1-related deafness subgroup over 18 months of observation has shown both follow-up interval, and pro-BDNF_0/BDNF_0 ratio play a significant role in the auditory development of the implanted children (Table 2). Of course, in the model we find that a follow-up interval, as in a regression model built using gene polymorphisms as predictors, is the most significant predictor*.* BDNF is a large molecule that is cleaved by proteases from pro-BDNF into the mature form and exerts a range of neurotrophic effects on neurons [36, 67]. Animal studies demonstrate that MMP-9 may also play a role in converting the pro-form of BDNF into its mature form [35]. Interestingly, recent accumulating evidence suggests that pro-BDNF and BDNF may have opposite effects on neuronal plasticity [33, 36, 67, 68]. Abnormalities in conversion of pro-BDNF to BDNF have already been postulated as helping to explain certain neuropathological processes underlying various brain disorders like bipolar depression or epilepsy [33, 35]. After applying the linear mixed-effect model methodology to our cohort of children with DFNB1-related deafness, we have found a progressive decrease in pro-BDNF_0/BDNF_0 ratio. Acting over the long neurodevelopmental process that occurs following a period of sensory deprivation, a change in ratio of 20 predisposes an implanted child to score 1 point higher in LEAQ (Table 2). However, we see a very wide range in the ratio of pro-BDNF_0/BDNF_0 in DFNB-1 related deafness subgroup (0.2 to 161.5, SD 29.4), so it is clear that the relation reflects a highly dynamic process.

### Perspective

Our data should be interpreted with caution. However, they do provide an indication of children’s capacity for successful speech and language rehabilitation after cochlear implantation. We have not isolated a biomarker of children who risk failure in speech and language rehabilitation, but we were able to point to a biochemical marker of good performers. Further research on larger sample size data and multicenter studies are needed to confirm this finding. However, broadening the preimplant diagnostic panel of commercially available ELISA (enzyme-linked immunosorbent assay) tests for MMP-9 plasma level would add considerable value and is easy to implement into clinical practice.

### Limitations

This is the first attempt to shed light on the molecular machinery of neuroplasticity after cochlear implantation in congenital deafness treatment, and as such it has many limitations and should be subject to wide-ranging critique. An undeniable weakness of this study is the measurement tool for assessing language development—the LEAQ and, in particular, its subjective character. Moreover, the effect of environmental factors, like parental/maternal educational status, the parents’ motivation to support the child through rehabilitation and the degree of speech training patients receive in it, is very difficult to control during such a study. We have followed the children only up to the 18^th^ month after CI activation; further longitudinal observation would add valuable detail on linguistic competency. Another factor limiting the group homogeneity, but which was out of our control, is the degree of cross-modal cortical reorganization before cochlear implantation. Despite a relatively short period of auditory deprivation in our patients, reorganization could have already taken place and could have affected the outcome [16, 69]. We used only a relatively small sample size, comprising subjects from the Polish population. Large-scale, multicenter studies incorporating subjects of different ethnicities are needed to see whether these results may be repeated more universally. Finally, both tested proteins are also involved in numerous other biological processes in the human body, which are not connected with neuronal plasticity, and so the relations between protein plasma level and phenotype seen in our material may not be repeatable in cases where there are other health problems.

## Supplementary Information

Below is the link to the electronic supplementary material.

Supplementary file1 (PDF 97 KB)

## Data Availability

The datasets analyzed during this study are accessible on reasonable request.
