# Supplementary material for: Prospective cohort study reveals MMP-9, a neuroplasticity regulator, as a prediction marker of cochlear implantation outcome in prelingual deafness treatment
Source: Mol Neurobiol. 2022 Jan 21;59(4):2190–203. doi: 10.1007/s12035-022-02732-7 (PMC9262127; doi:10.1007/s12035-022-02732-7)
Supplement: Supplementary file 1 — Supplementary file1 (PDF 97 KB) [file 12035_2022_2732_MOESM1_ESM.pdf]

Supplementary Tables

Prospective cohort study reveals MMP-9, a neuroplasticity regulator, as a prediction marker of cochlear implantation outcome in prelingual deafness treatment.

Monika Matusiak*, Dominika Oziębło*, Monika Ołdak, Emilia Rejmak, Leszek Kaczmarek, Piotr Henryk Skarżyński, Henryk Skarżyński

Corresponding author: Monika Matusiak, Oto-Rhino-Laryngosurgery Clinic, Institute of Physiology and Pathology of Hearing, M. Mochnackiego 10, 02-042 Warsaw, Poland, tel. +48 22 356 0366, mail: m.matusiak@ifps.org.pl

*These authors contributed equally to this work.

Supplementary Table 1. Numbers and frequencies of alleles reported in the present study and population databases.

RefSNP number	Alleles	Present study		gnomAD (EUR non-Finnish)		*p*-value	1000 Genomes (EUR)		*p*-value
		Number	Frequency	Number	Frequency		Number	Frequency	
rs3918242	C	104	0.852	123528 (55087)	0.864 (0.854)	0.708 (1)	4231 (832)	0.845 (0.827)	0.823 (0.823)
	T	18	0.148	19460 (9439)	0.136 (0.146)		777 (174)	0.155 (0.173)	
rs20544	C	61	0.500	80694 (461)	0.564 (0.458)	0.152 (0.383)	3709 (27369)	0.741 (0.424)	<0.0001 (0.092)
	T	61	0.500	62304 (545)	0.436 (0.542)		1299 (37109)	0.259 (0.576)	
rs2234681	<20	68	0.557	N/A			N/A		
	≥20	54	0.443						
rs6265	Val	104	0.852	122202 (52463)	0.853 (0.813)	1 (0.264)	4000 (808)	0.799 (0.803)	0.143 (0.191)
	Met	18	0.148	20988 (12077)	0.147 (0.187)		1008 (198)	0.201 (0.197)	

N/A – not available

Supplementary Table 2. Relations between mean values of plasma levels of MMP-9 and BDNF proteins collected at cochlear implantation and the genes polymorphisms in the study group.

		plasma level (ng/ml) , SD	p-value
***MMP9* rs3918242**			
	CC (43) /CT (18)	210.5 (105) / 299 (178.3)	0.07
***MMP9* rs2234681**			
	<20/<20 (17) >-20/≥20(10)	217.3 (108.5) / 229.6 (149.2)	0.82
	<20/<20 (17) <20/≥20 (34)	217.3 (108.5) / 248.9 (145.9)	0.61
	<20/≥20 (34) ≥20/≥20(10)	248.9 (145.9) / 229.6 (149.2)	0.59
***MMP9* rs20544**			
	C/T(29) T/T(16)	236.1 (109.1) / 217 (113.4)	0.58
	C/C(16) T/T(16)	258.4 (193.8) / 217 (113.4)	0.89
	C/C(16) C/T(29)	258.4 (193.8) / 236.1 (109.1)	0.78
***BDNF* rs6265**			
	Val/Val(43) Val/Met(18)	2 (1.4) / 2.9 (2.8)	0.36

Supplementary Table 3. Relations between mean values of plasma levels of MMP-9 and BDNF proteins collected at cochlear implantation and the genes polymorphisms in the DFNB1-related deafness subgroup.

		plasma level (ng/ml), SD	p-value
***MMP9* rs3918242**			
	CC (30) /CT (10)	203.9 (101.2) / 282.4 (107.9	0.06
***MMP9* rs2234681**			
	<20/<20 (11) >-20/≥20(6)	222.5 (110) / 172.4 (98.3)	0.3
	<20/<20 (11) <20/≥20 (23)	222.5 (110) / 237 (108.4)	0.7
	<20/≥20 (23) ≥20/≥20(6)	237.4 (108.4) / 172.4 (98.3)	0.1
***MMP9* rs20544**			
	C/T(21) T/T(11)	239.4 (109.8) / 216(114.2)	0.5
	C/C(8) T/T(11)	192.4 (94.6) / 216 (114.2)	0.6
	C/C( ) C/T( )	192.4 (94.6) / 239.4 (109.8)	0.2
***BDNF* rs6265**			
	Val/Val(29) Val/Met(11)	2.3 (1.6) / 3.2 (3.3.)	0.6

Due to small number of tested genotypes (for example 2 carriers of ≥20/≥20 *MMP9* rs2234681in the younger group) we do not present results of the analysis in subgroups age at CI activation up to and after 1 year of life.
